# Anticancer Activity of Ethanolic Extract of *Tabernaemontana catharinensis* in Breast Cancer Lines MCF-7 and MDA-MB-231

**DOI:** 10.3390/ijms26168111

**Published:** 2025-08-21

**Authors:** Diana del Carmen Martínez-Méndez, María de la Luz Sánchez-Mundo, María del Rocío Thompson-Bonilla, Luis Marat Álvarez-Salas, Víctor Hugo Rosales-García, Jacobo Rodríguez-Campos, María Eugenia Jaramillo-Flores

**Affiliations:** 1 Ingeniería Bioquímica, Escuela Nacional de Ciencias Biológicas (ENCB)-Instituto Politécnico Nacional, Ciudad de México 07738, Mexico; dmartinezm2111@alumno.ipn.mx; 2 ITS de Las Choapas, Tecnológico Nacional de México, Carretera Las Choapas-Cerro de Nanchital Km 6.0, Col. J. Mario Rosado, Las Choapas 96980, Mexico; l-sanchezm@choapas.tecnm.mx; 3 Laboratorio de Medicina Genómica del Hospital Regional “1° de Octubre” del Instituto de Seguridad y Servicios Sociales de los Trabajadores del Estado (ISSSTE), Av. IPN 1669, Col. Magdalena de las Salinas, Ciudad de México 07760, Mexico; maria.thompson@issste.gob.mx; 4 Centro de Investigación de Estudios Avanzados del Instituto Politécnico Nacional (CINVESTAV), Ciudad de México 07360, Mexico; lalvarez@cinvestav.mx (L.M.Á.-S.); vrosales@cinvestav.mx (V.H.R.-G.); 5 Unidad de Servicios Analíticos y Metodológicos, Av. Normalistas 800, Col. Colinas de la Normal, Guadalajara 44270, Mexico; jarodriguez@ciatej.mx

**Keywords:** MCF-7, MDA-MB-231, cell cycle, CDK4, ALDH3A1, p53, apoptosis, invasion, combinations

## Abstract

Breast cancer is a serious public health problem worldwide. Although current treatments with drugs such as cisplatin and paclitaxel are effective, they are associated with severe adverse effects and the development of drug resistance, which has prompted the search for new therapeutic strategies. In this context, the present study evaluated the anticancer activity of the ethanolic extract of *Tabernaemontana catharinensis* (EET) on the breast cancer cell lines MCF-7 (hormone-sensitive) and MDA-MB-231 (triple-negative) using 2D and 3D models. The results showed that EET significantly reduced cell viability in both lines, with IC_50_ values of 83.06 µg/mL (MCF-7) and 8.3 µg/mL (MDA-MB-231) in 2D and 499.3 µg/mL and 280 µg/mL, respectively, in 3D. In addition, treatment with EET caused cell cycle arrest in the G1 phase, reduced CDK4 activity by 58% and ALDH3A1 activity by 32%, and increased levels of the tumor suppressor protein p53. Significant induction of apoptosis was also observed, evidenced by the activation of caspases -3/7, -8, and -9, along with a decrease in intracellular ATP levels (37% in MCF-7 and 90% in MDA-MB-231), suggesting mitochondrial dysfunction. Finally, EET showed the ability to inhibit cell invasion. Taken together, these results indicate that the ethanolic extract of *Tabernaemontana catharinensis* has potent antiproliferative, proapoptotic, and antimetastatic activity in breast cancer cells, in both two-dimensional and three-dimensional models. Its effect on various key molecular pathways and its ability to enhance the action of conventional chemotherapeutic agents position it as a promising adjuvant agent in the treatment of breast cancer.

## 1. Introduction

Breast cancer is one of the most widely recognized cancers worldwide [1], ranking second in incidence only after lung cancer, and continues to increase [2], making it imperative to develop effective therapies. It is a disease with different subtypes characterized by unique molecular patterns [3]. The study of cell lines such as MCF-7 (ER+ estrogen receptor cell line) and MDA-MB-231 (triple-negative cell line) allows for the expansion of findings in breast cancer due to the molecular characteristics of each [4]. There are different treatments for this disease, including surgery, radiation therapy, targeted therapy, endocrine therapy, immunotherapy, and the most widely used, chemotherapy [5], which includes the use of antineoplastic drugs such as cisplatin and paclitaxel [6,7]. However, these drugs have high and varied adverse effects [8]. Today, treatments that act on specific therapeutic targets are being sought, and biomarkers such as ER (estrogen receptor), PR (progesterone receptor), and HER-2 (human epidermal growth factor receptor 2) are evaluated when deciding on the appropriate treatment for the patient [9].

The three main subtypes of tumors are (a) positive hormone receptors, (b) HER-2 overexpression, and (c) without defined molecular targets, also known as triple-negative tumors. Therefore, they have different treatment characteristics: (1) CDK4/6 inhibitors (Palbociclib) are used in hormone receptor-positive breast cancer [10]; (2) treatments such as Trastuzumab are used in neoplasms with HER-2 overexpression [11]; (3) for triple-negative tumors, immunotherapy is used, which includes drugs such as Pembrolizumab, a monoclonal antibody targeting programmed cell death protein 1 (PD-1) [12]. However, due to the adverse side effects of antineoplastic drugs, there is a constant search for new therapeutic alternatives. Among these, secondary plant metabolite anticarcinogens show great potential for developing new drugs [13].

*Tabernaemontana catharinensis* belongs to the Apocynaceae family and is widely distributed in tropical and subtropical regions. Higashi et al. [14] extracted different alkaloids (Ibogamine, coronaridine, Na-methyl-Pericyclivine (C-SA), Tabernanthine Vocangine, Affinisine, isomer of Voachalotine, Isovoacangine, Conopharinginine, Voachalotine, Na-methyl-polyneuridine aldehyde ¼ Dehydro-voachalotine (C-AS), Isoconopharinginine, 11-Methoxy-voachalotine (C-AS)) from the latex of this plant with hexane. Rizo et al. [15] provided evidence that compounds from *Tabernaemontana catharinensis* have cytotoxic effects on human laryngeal epithelial carcinoma cells (Hep-2) induced by coronaridine, an alkaloid isolated from the plant. The study indicated that coronaridine’s ability to induce apoptosis was comparable to that of established chemotherapeutic agents, suggesting its potential as a natural compound for cancer therapy. Boligón et al. [16] highlight that the compounds present in this plant have antitumor efficacy. Sari et al. [17] identified that this plant contains quercetin, rutin, ferulic acid, coumaric acid, and pinocembrin, all of which are phenolic compounds when extracted in ethanol.

Phenolic compounds are a good alternative in cancer treatment [18]. Between them, phenolic acids such as caffeic acid, found in some fruits, coffee, and certain vegetables, have anticancer, anti-inflammatory, antimicrobial, immunoregulatory, and antioxidant potential [19], and protocatechuic acid shares these characteristics [20]. These compounds have demonstrated antitumor properties in breast cancer cell lines (MCF-7, MDA-MB-231), significantly reducing cell viability [21]. This study evaluated cytotoxicity, proapoptotic activity, cell cycle arrest, and invasion inhibition in MCF-7 and MDA-MB-231 breast cancer cell lines in 2D and 3D models of *Tabernaemontana catharinensis* ethanolic extract.

## 2. Results

### 2.1. Compounds Identification of EET by Ultra-Performance Liquid Chromatography with Time-of-Flight Mass Spectrometry (UPLC-Q-TOF/MS^E^) and Quantification of Compounds in Crude Extract by HPLC

The plant material was provided by a local plant supplier and transported in bags. Handling was carried out under aseptic conditions (disinfected surfaces). The samples were then labeled and stored for further processing. Extracts were made from these samples for analysis. Table 1 lists the compounds identified in the extract, which are caffeic acid 3-glucoside, protocatechuic acid, protocatechuic acid 4-glucoside, chlorogenic acid, caffeic acid, and coniferyl aldehyde. By HPLC, 1.2 mg chlorogenic acid/g dry sample represents 84.3% of the quantified phenols, 118 µg caffeic acid/g dry sample (8.3%), and 105 µg protocatechuic acid/g dry (7.4%) sample were found (Table 2). Caffeic and protocatechuic acids have reducing and chelating capacities and support antioxidant and anti-inflammatory activities observed in cellular and animal models [22]; chlorogenic acid also exhibits antioxidant, anti-inflammatory, and antiproliferative effects [23], and these compounds have anticancer properties [24,25].

### 2.2. Two-Dimensional Cell Model Assays

#### 2.2.1. EET Decreases MCF-7 and MDA-MB-231 Cell Viability

**Figure 1:**
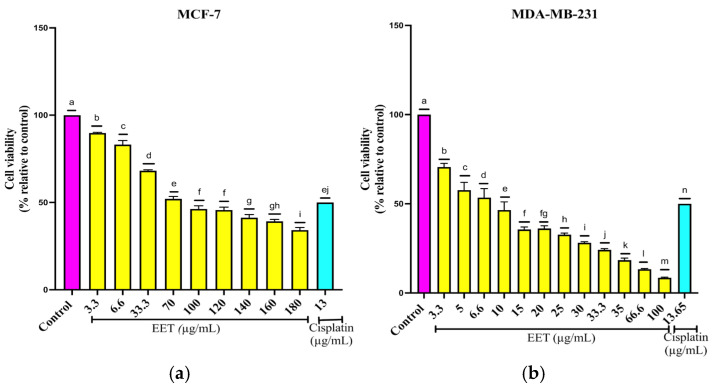
Reduction in the viability of breast cancer cells in a 2D model by EET; (**a**) MCF-7, (**b**) MDA-MB-231. Results are expressed as mean ± SD values. *p* < 0.0001 according to Tukey’s post hoc test, *n* = 12. Different letters represent significant differences between types of treatment.

The EET decreases cell viability in both lines, having an IC_50_ of 83.06 µg/mL for MCF-7 and 8.3 µg/mL for MDA-MB-231 (Figure 1). The results demonstrated that higher concentrations of EET led to increased cell death, indicating a dose-dependent effect.

#### 2.2.2. Cell Cycle Arrest in the G1 Phase by EET Treatment in a 2D Model of MCF-7 and MDA-MB-231 Cells

The cell cycle is a dynamic and versatile mechanism that is involved in the pathophysiology of several human systems. It is characterized by several checkpoints, especially in the transitions from G1 to S phase and from G2 to M phase [26]. Cellular stress can lead to cell cycle inhibition through checkpoint activation. G1/S phase checkpoint control prevents replication of damaged DNA, while G2/M phase checkpoint control prevents segregation of damaged chromosomes into daughter cells during mitosis [27]. In neoplastic cells, these controls are disrupted, so cells divide and proliferate uncontrollably [28], which is why cell cycle arrest in cancer cells is of vital importance.

Based on the results of cell cycle arrest, where it was found that EET stops the cycle in the G1 phase, one of the proteins that executes this phase is CDK4. Therefore, we analyzed the effect of EET directly on the protein. As shown in Table 3, EET arrests the cell cycle in G1 phase at both 500 and 1000 µg/mL in both cell lines; in MCF-7 in G1 phase has 70.3%, in S 0.77% and in G2 28.2% at 500 µg/mL; while the distribution at 1000 µg/mL is in G1 86.1%, S 13.24% and G2 0.66%; for MDA-MB-231, it is in G1 97.7%, S 2.3%, and 0% in the G2 phase at 500 µg/mL, G1 100%, S 0%, and G2 0% at 1000 µg/mL.

#### 2.2.3. Decrease of Human Aldehyde Dehydrogenase 3A1 (ALDH3A1) Activity by EET Treatment

**Figure 2:**
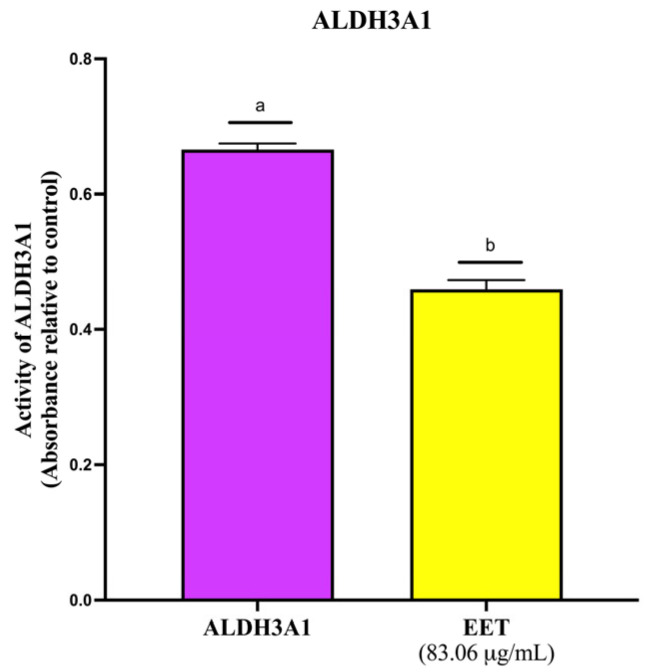
Reduction in ALDH3A1 activity due to treatment with 83.06 µg/mL of EET. Results are expressed as mean ± SD values. *p* < 0.0015 according to Tukey’s post hoc test, *n* = 6. Different letters represent significant differences between types of treatment.

Aldehyde dehydrogenases are a group of NAD+ dependent enzymes [29,30]. They are divided into 11 families and have 19 members in total: ALDH1 (1A1, 1A2, 1A3, 1B1, 1L1, and 1L2), ALDH2, ALDH3 (3A1, 3A2, 3B1, and 3B2), ALDH4A1, ALDH5A1, ALDH6A1, ALDH7A1, ALDH8A1, ALDH9A1, ALDH16A1, and ALDH18A11 [31]. To determine whether part of the mechanism of EET-induced cell death involves inhibition of ALDH3AI, the inhibition of the enzyme by the extract was quantified. As shown in Figure 2, the EET decreases ALDH3A1 enzyme activity by 32% compared to the control. This finding is particularly relevant, as recent studies have demonstrated that overexpression of this protein is associated with poor prognosis in various types of cancer, including breast cancer [32].

#### 2.2.4. Decrease in Cyclin-Dependent Kinase 4 (CDK4) Activity by EET Treatment

**Figure 3:**
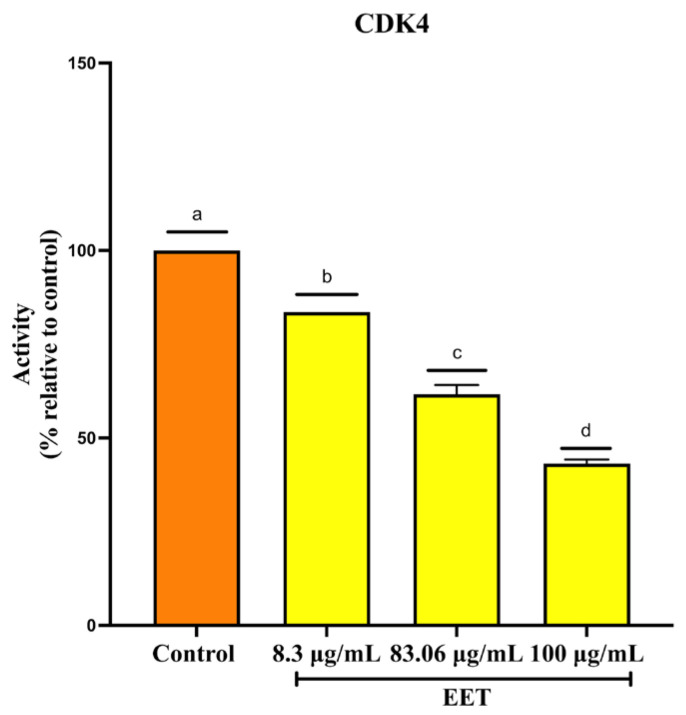
Reduction in cyclin-dependent kinase 4 (CDK4) activity by EET. Results are expressed as mean ± SD values. *p* < 0.0001 according to Tukey’s post hoc test, *n* = 6. Different letters represent significant differences between types of treatment.

Cell cycle progression requires a series of tightly regulated events mediated by the expression and interaction of various cyclin-dependent kinases (CDKs) with the corresponding cyclins [33]. CDK4 forms a complex with cyclin D, which drives progression through the G1 phase, a critical stage during which the cell prepares for DNA synthesis [34]. Based on the results of cell cycle arrest, where it was discovered that EET stops the cycle in the G1 phase, the study of CDK4, a protein involved in this phase, was continued. Treatment with EET decreased CDK4 activity by 17% at 8.3 µg/mL, 40% at 83 µg/mL, and 58% at 100 µg/mL, the highest concentration tested (Figure 3).

#### 2.2.5. Increase in p53 Protein Activity by EET Treatment

**Figure 4:**
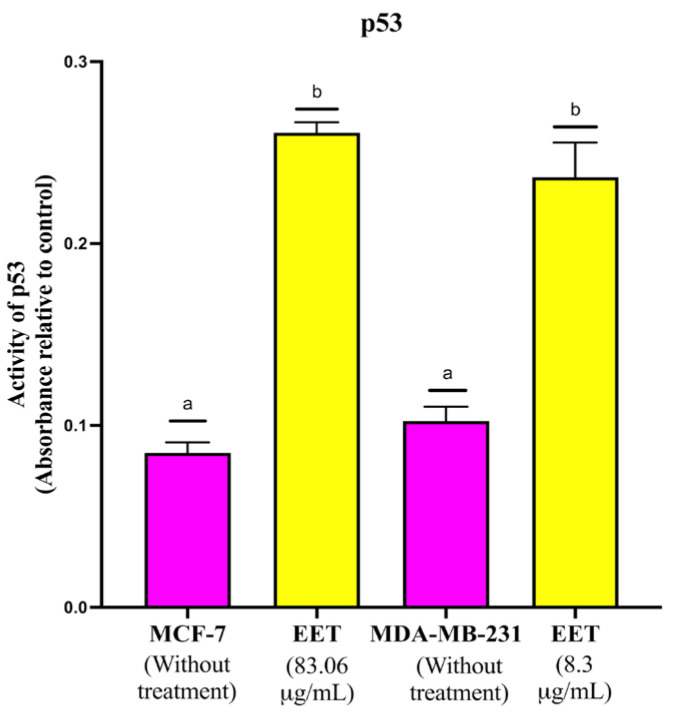
Increase in p53 activity by EET in nuclear extracts from MCF-7 and MDA-MB-231 cell lines. Results are expressed as mean ± SD values. *p* < 0.0001 according to Tukey’s post hoc test, *n* = 6. Different letters represent significant differences between types of treatment.

The p53 protein is a transcription factor that controls the outcome of numerous biological processes depending on the type of cellular stress signal. The stress signals that activate p53 are activation of oncogenes, DNA damage, and replicative stress [35]. p53 is involved in important and decisive biological processes for the cell such as cell cycle arrest, senescence, DNA repair, and apoptosis [36]. As can be seen in Figure 4, p53 activity is increased with EET in both cell lines. The same increase in p53 activity in MDA-MB-231 is achieved at a tenfold lower EET concentration than for MCF-7.

### 2.3. Three-Dimensional Cell Model Assays

#### 2.3.1. EET Decreases Cell Viability in 3D Models by Assessing Plasma Membrane Integrity in MCF-7 and MDA-MB-231 Cells

**Figure 5:**
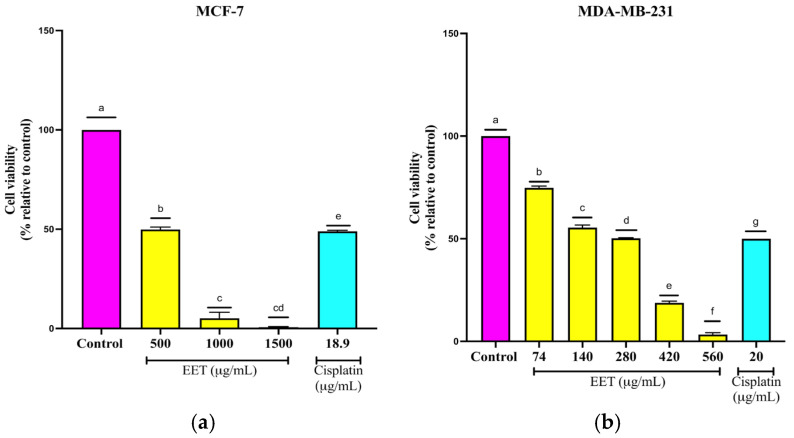
Reduction in the viability of breast cancer cells in a 3D model by EET; (**a**) MCF-7, (**b**) MDA-MB-231. Results are expressed as mean ± SD values. *p* < 0.0001 according to Tukey’s post hoc test, *n* = 6. Different letters represent significant differences between types of treatment.

EET decreased cell viability in MCF-7 cell lines, 50% at 500 µg/mL, 96% at 1000 µg/mL, and 100% at 1500 µg/mL (Figure 5a). In the MDA-MB-231 cell line, viability decreased 25, 45, 50, 50, 81, and 97% at concentrations of 74, 140, 280, 420, and 560 µg/mL, respectively (Figure 5b). These findings demonstrate the cytotoxicity of the bioactive compounds present in the extract on breast cancer cell lines.

#### 2.3.2. EET Decreases ATP Levels of MCF-7 and MDA-MB-231 Cells in the 3D Model

**Figure 6:**
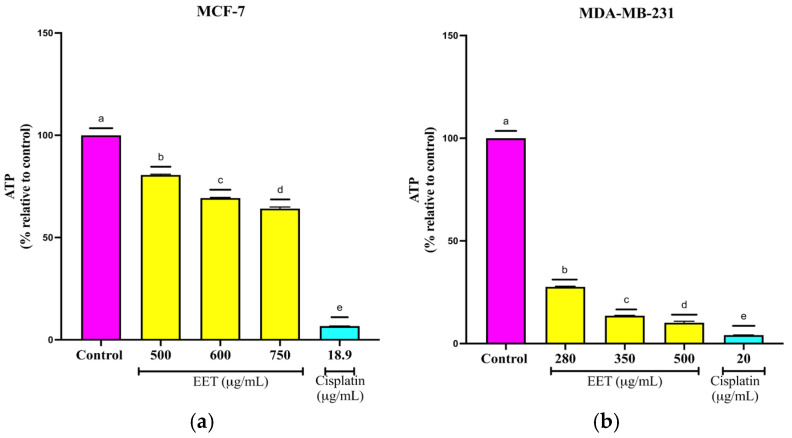
Decrease in ATP levels in breast cancer cells in a 3D model treated with EET; (**a**) MCF-7, (**b**) MDA-MB-231. Cell viability determined by ATP level quantification. Results are expressed as mean ± SD values. *p* < 0.0001 according to Tukey’s post hoc test, *n* = 6. Different letters represent significant differences between types of treatment.

Intracellular ATP is essential for cell survival and the proper functioning of cellular components, including proliferation in both malignant and non-terminally differentiated immune cells) [37]. As shown in Figure 6a, treatment with EET reduced ATP levels 20% at 500, 30% at 600 µg/mL, and 37% at 750 µg/mL; in MDA-MB-231 cells, ATP levels were reduced by 72% at 280, 86% at 350 µg/mL, and 90% at 500 µg/mL (Figure 6b).

#### 2.3.3. EET Activates Caspases -3/7, -8 and -9 in MCF-7 and MDA-MB-231 Cell Lines in a 3D Model

Activation of caspases is a central event in apoptotic signaling and can be triggered through either the intrinsic (mitochondrial) or extrinsic (death receptor) pathways. Caspases are broadly classified as initiator caspases, including -8 and -9, and effector caspases such as -3, -6, and -7 [38].

**Figure 7:**
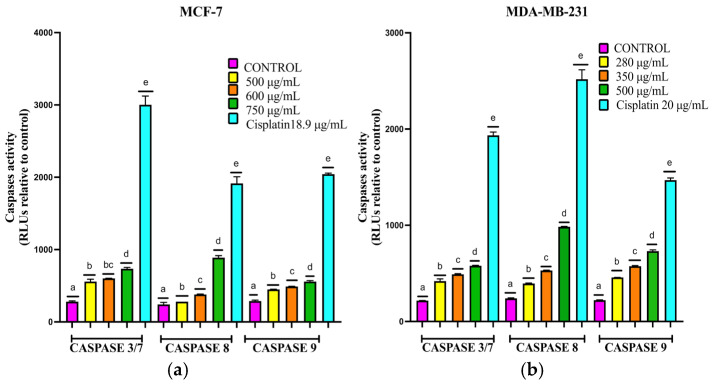
(**a**) Changes in the activity of initiator and effector caspases in apoptosis in MCF-7 cells in a 3D model treated with EET. Apoptosis is induced by the activity of caspases -3/7, -8, and -9. Spheroids were exposed to 500, 600, 750 µg/mL of EET and 18.9 µg/mL of cisplatin for 24 h. (**b**) Changes in the activity of initiator and effector caspases in apoptosis in MDA-MB-231 cells in a 3D model treated with EET. Apoptosis is induced by caspase-3/7, -8, and -9 activity. Spheroids were exposed to 280, 350, 500 µg/mL of EET and 20 µg/mL of cisplatin for 24 h. Mean values and standard deviation of relative fluorescence units by Tukey’s test of a posteriori comparison (*p* ≤ 0.0001), *n* = 6. Different letters represent significant differences between treatment types.

As shown in Figure 7a, 24 h treatment of MCF-7 spheroids with EET (500, 600, and 750 µg/mL) markedly increased the activities of caspase-3/7, -8, and -9. Caspase-8 exhibited the greatest relative activation, although its levels remained below those produced by the positive control (18.9 µg/mL-cisplatin). Maximal activation for all three caspases occurred at 750 µg/mL EET Similarly, in Figure 7b, MDA-MB-231 spheroids exposed to EET (280, 350, 500 µg/mL displayed elevated activities of caspases -3/7, -8, and -9, albeit to a lesser extent than those treated with 20 µg/mL-cisplatin. As in MFC-7 cells, caspase-8 was the most strongly activated protease.

#### 2.3.4. Inhibition of Cell Invasion by EET in MCF-7 and MDA-MB-231 Cells in a 3D Model

Inhibiting tumor-cell invasion is a critical therapeutic goal, as invasion represents one of the earliest steps in the metastatic cascade [39]. Metastasis—colonization of distant organs by cancer cells—is the leading cause of cancer mortality [40]. Cellular plasticity, defined as the ability of cells to adopt new phenotypic states, underpins tumor initiation and progression by enabling high proliferative and metastatic potential [41].

**Figure 8:**
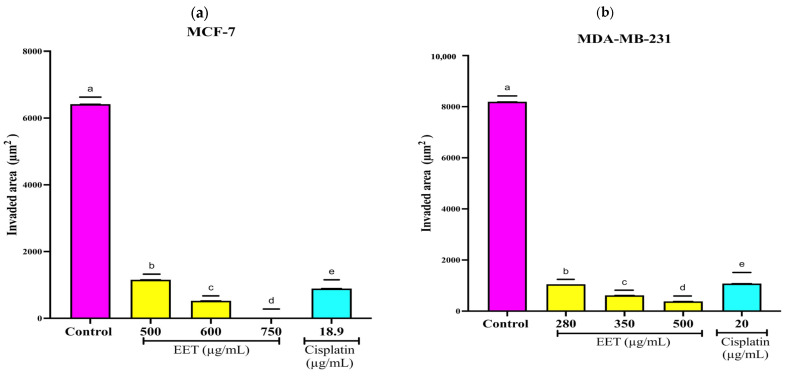

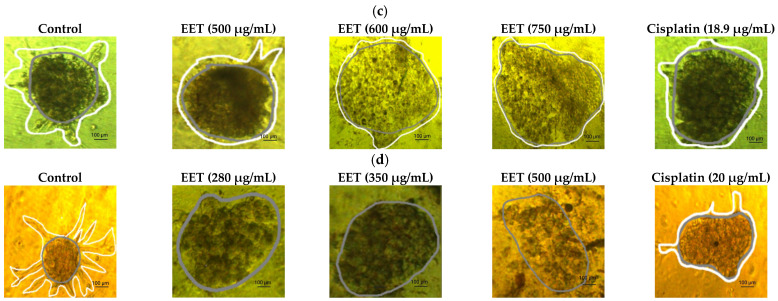
EET inhibits the invasive capacity of MCF-7 and MDA-MB-231 cells in a three-dimensional system. Tumor invasion was evaluated based on the area and perimeter occupied within the matrigel. Data are expressed as means ± standard deviation of relative fluorescence units and were analyzed using Tukey’s post hoc test (*p* ≤ 0.0001; *n* = 6). (**a**) Invaded area (μm^2^) in MCF-7 cells. Different letters indicate statistically significant differences between treatments. (**b**) Invaded area (μm^2^) in MDA-MB-231 cells. Different letters indicate significant differences between treatment groups. (**c**) Micrographs of MCF-7 spheroids obtained by inverted microscopy (20×, scale 100 μm). The gray line delimits the edge of the spheroid, while the white line indicates the extent of the invading cells. The invaded area corresponds to the zone between the two lines. (**d**) Micrographs of MDA-MB-231 spheroids recorded with inverted microscopy (20×, scale 100 μm). The gray line indicates the contour of the spheroid and the white line marks the limit of cell invasion. The invaded area was defined as the space between both boundaries.

As illustrated in Figure 8a, EET reduced the invasion of MFC-7 spheroids by 82% at 500 µg/mL, by 92% at 600 µg/mL, and by 100% at 750 µg/mL compared to the control. In the MDA-MB-231 line, 87% at 280 µg/mL, 92% at 350 µg/mL, and 96% at 500 µg/mL concerning the control (Figure 8b).

#### 2.3.5. EET in Co3mbination with Cisplatin and Paclitaxel Decreases the Viability of MCF-7 and MDA-MB-231 in 2D and 3D Models

As shown in Table 4, the combination of the mean inhibitory concentration of cisplatin with EET in the 2D model significantly improved cytotoxicity, achieving 70% inhibition for MCF-7. In MDA-MB-231, when half the IC_50_ of EET and the IC_50_ of cisplatin are used, there is synergy, and an additive effect is shown when the respective IC_50_ of the drug and the extract are combined. The combination of paclitaxel with EET showed significant inhibition results, with a synergistic effect of the combination of half the IC_50_ of EET and the IC_50_ of the antineoplastic and the mixture of the respective IC_50_s of both in MCF-7. In MDA-MB-231, the combinations of half the IC_50_ of both and half the IC_50_ of the extract with the IC_50_ of paclitaxel show a synergistic effect, and the combinations of the IC_50_ of EET and half the IC_50_ of the drug and the combination of the respective IC_50_s show an additive effect. In the 3D model, an additive effect was only shown when half of the IC_50_ of the extract and the IC_50_ of paclitaxel were combined in the MCF-7 cell line (Table 5), using the Chou–Talalay methodology [42] (Table S1).

## 3. Discussion

Current research has focused on the search for phytochemicals that can be used in cancer treatment. The present study shows the cytotoxic effect of an ethanolic extract of the plant *Tabernaemontana catharinensis* (EET) on breast cancer cell lines (MCF-7; MDA-MB-231-231).

*Tabernaemontana catharinensis* is a plant that exhibits significant biological activity due to its composition. In an in vivo study, treatment with *Tabernaemontana* at a concentration of 10 μg/ear reduced ear edema and MPO (myeloperoxidase) by 100% and 94%, respectively, and also reduced the levels of proinflammatory cytokines (MIP-2, IL-1β, and TNF-α) [43].

Boligon et al. [16] evaluated the composition and antioxidant capacity of the crude extract and fractions of *Tabernaemonatana catharinensis* using HPLC/DAD and GC/MS and found that this species contains gallic, chlorogenic, and caffeic acids, as well as rutin, quercetin, and kaempferol.

Rosales et al. [44] identified indole alkaloids (16-epi-afinina, 12-methoxy-n-methyl-voachalotina, afinisina, voachalotina, coronaridina hidroxiindolina, and ibogamina), which were subjected to an in silico toxicity test using the “ADME -Tox” and OSIRIS software, and also, molecular docking using topoisomerase I (PDB ID: 1SC7) using iGEMDOCK showed that the fraction with afinisine exhibited selective toxicity against A375 (skin cancer cells) with an IC_50_ of 11.73 µg/mL and no cytotoxicity against normal cells (Vero).

On the other hand, Boligon, Schwanz et al. [45] analyzed the composition of the essential oil (β-caryophyllene (56.87%), α-cadinol (12.52%), 8S,13-cedran-diol (5.41%), α-terpineol (3.99%), β-eudesmol (2.54%), caryophyllene oxide (2.51%), and ethyl isoalocolate (2.03%), along with β-cubebene, γ-cadinene, cubenol, 1,8-cineole, o-cymene, curcumenol, spatulenol, friedelin, and β-sitosterol) from this catharinensis plant using gas chromatography–mass spectrometry (GC-MS). Furthermore, in a study conducted by da Silva Menecucci et al. [46], they found alkaloids in the latex. Piana et al. [47] used the DDPH technique to demonstrate that the compounds in this plant (phenolic compounds) have good antioxidant capacity.

EET decreased the viability of both cell lines in 2D and 3D models. This can be attributed to compounds present in the extract such as protocatechuic and chlorogenic acids, which inhibit proliferation in MCF-7 cells by increasing DNA fragmentation and decreasing mitochondrial membrane potential [48], which are markers of cell death [49,50]. Caffeic acid has been shown to decrease breast cancer cell proliferation [19], and chlorogenic acid is cytotoxic in ductal (MCF-7) and metastatic (MDA-MB-453) breast cancer cells [51].

MDA-MB-231 cells are ten times more sensitive to this treatment, probably due to the presence of caffeic acid, which is known to act on FOXO1 (Forkhead Box O1), decreasing levels in triple-negative breast cancer [52] FOXO1 increases the probability of forming breast cancer stem cell spheres and simultaneously induces substantial accumulation of SOX2 (sex-determining region Y-box protein 2) [53], which allows cells to differentiate and evolve into other cell types and is responsible for the ability to grow tumor cells [54]. A lower expression of FOXO1 implies lower cell survival, lower recurrence, and decreased metastasis [55]. This may be due to the chlorogenic acid present in the extract, which lowers ATP levels in breast cancer cells, in addition to lowering adenosine levels [56], which is a metabolic regulator that relates energy status to processes such as immunomodulation and cell proliferation. Tumors create an adenosine-rich microenvironment through the increased release of ATP from senescent and stressed cells and its ectoenzymatic conversion to adenosine [57].

The compounds present in the extract cause the cells not to continue the progression through the cycle and thereby stop proliferation. Rosendahl et al. [58] tested caffeic acid in breast cancer cell lines (MCF-7, T47D, and MDA-MB-231), demonstrating that caffeic acid inhibits cell proliferation by modulating ER (estrogen receptor -a) and IGFIR (IGF type I receptor) levels, influencing cell cycle progression. These authors found that caffeic acid acts best on MCF-7 and is reported to arrest the cell cycle in the G1 phase in colon cancer and glioblastoma [59]. In this work (Table 3), the best results were obtained with MDA-MB-231 at 1000 µg/mL, since cell arrest occurs entirely in the G1 phase. This may be due to the joint action with chlorogenic acid present in the extract, probably because this compound arrests the cell cycle in the G0/G1 phase due to the increase of p21 and p53 proteins [29].

In the cell cycle assay, higher concentrations of the extract are needed due to its complexity, since it contains compounds with different bioavailability, and higher concentrations are needed to achieve the desired biological effect. A wide variety of studies start with high concentrations, even though the IC_50_ is lower. Pumiputavon et al. [60] used concentrations of 500–1000 µg/mL of methanol extracts from *Annonaceae* plants in several cell lines (HepG2, Hep3B, K562, U937, and RAJI) to observe changes in the cell cycle. Chinnasamy et al. [61] used concentrations of 600–1000 µg/mL of ethanol extract from *Mucuna pruriens* seeds.

Aldehyde dehydrogenase 3A1 (ALDH3A1) is an enzyme that oxidizes various endogenous and exogenous aldehydes to carboxylic acids. TSA decreased the activity of this enzyme, and depletion of this protein has been shown to decrease ATP levels and induce apoptosis. In addition, low ALDH3A1 activity increases drug cytotoxicity [62]. In preclinical models to analyze sensitivity to drugs such as cyclophosphamide and other oxazaphosphorines (4-hydroxycyclophosphamide, mafosfamide, and ifosfamide) [63], drugs used in the palliative treatment of advanced breast cancer [64]. ALDH3A1 is studied because this enzyme detoxifies these neoplastic agents and reduces their effectiveness [63]. Consequently, reducing enzyme activity will increase the efficacy of drugs.

Voulgaridou et al. [65] demonstrated that increased expression of ALDH3A1 in MCF-7 cells increased levels of EpCAM (CD326; epithelial cell adhesion molecule) and CD49f (integrin a6), proteins that increase aggressiveness and metastasis in breast cancer [66].

**Figure 9:**
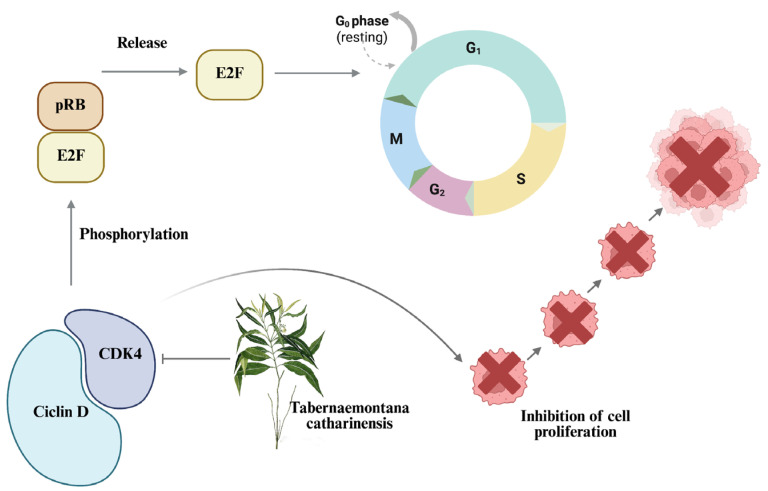
EET-mediated CDK4 inhibition mechanism. The binding of CDK4 (cyclin-dependent kinase 4) with cyclin D hyperphosphorylates pRB (retinoblastoma), releasing the E2F complex (transcription factor 2), so that it can carry out the transcription of proteins required for the S phase. In the presence of EET, CDK4 is inhibited, which prevents the formation of the complex with cyclin D, thereby inhibiting cell proliferation.

CDK4 activity was decreased by more than 50%. This is relevant because if CDK4 is inactivated or lowers its levels, it cannot bind to cyclin D, thus stopping cell cycle progression. This complex is responsible for hyperphosphorylating pRB and its related proteins p107 and p130 [67], thus allowing the release of the E2F complex, responsible for activating the transcription of genes that will be used in S phase [68] and therefore progressing the cell cycle (Figure 9). Liang et al. [69] performed a study with caffeic acid showing cell cycle arrest of nasopharyngeal cancer in G1 by CDK4 downregulation.

In addition, an increase in p53 protein is shown. This may be due to the fact that the compounds present in the extract, such as caffeic acid and phenethyl ester of caffeic acid, has the ability to overexpress p53 in breast cancer cell lines (MCF-7 and MDA-MB-231). Rezaei-Seresht et al. [19] observed that caffeic acid inhibits the growth of triple-negative cells because it negatively regulates mortalin [70], a positively regulated protein in the most aggressive cancer cells such as MDA-MB-231, and its downregulation leads to a significant increase in cell death [71]. On the other hand, chlorogenic acid has been shown to induce apoptosis by up-regulating p53 and thereby tumor suppression [72,73].

The transition from two-dimensional (2D) cell cultures to three-dimensional (3D) spheroid models in cancer research represents a significant advance in tumor modeling fidelity. This transition requires consideration of the concentration of compounds used in 3D models compared to 2D models, due to the more complex microenvironment simulated by 3D spheroids.

The IC_50_ of the 3D model was approximately 6 times higher in MCF-7 and 30 times higher in MDA-MB-231. The increase in concentrations is attributed to the in vivo-like morphological complexity, variable polarity, and multi-plane adhesion of spheroid structures. Kabalan et al. [74] noted that 3D cancer cell spheroids better replicate the tumor microenvironment, including oxygen gradients and interactions with the extracellular matrix (ECM). These conditions often require higher doses of anticancer compounds to achieve therapeutic effects comparable to those that can be achieved with lower concentrations in 2D cultures. This is due to the greater resistance observed in 3D cultures, where cells can exhibit adaptive mechanisms to survive and grow within their more natural environment.

This type of model has the advantage of providing an outline of the behavior of compounds and is necessary for clinical trials [75]. However, this type of test still has certain disadvantages, such as being static and not capturing distribution/metabolism at the organism level [76], and in vivo tumors have albumin, fibrinogen/fibrin, and other plasma proteins in the tumor interstitial fluid (TIF) [77] and accumulate hyaluronan, tenascin-C, periostin, collagen I/III [78], which do not exist in spheroids.

It is shown that the extract activates caspases -3/7, -8, and -9. This suggests that the compounds present are activating these proteins. Yin et al. [48] conducted an investigation in which it was observed that protocatechuic acid can increase the levels of caspase-8 and -3. In addition, in both cell lines, activation of caspase-9 can be seen; this may be due to caffeic acid, which activates caspases and interacts with the p44/42 MAPK (Mitogen-activated protein kinase) complex [79]. p44/42 MAPK plays a complex role in apoptosis by influencing proapoptotic processes [80]; inhibition of this protein potentiates apoptosis in breast cancer [81].

**Figure 10:**
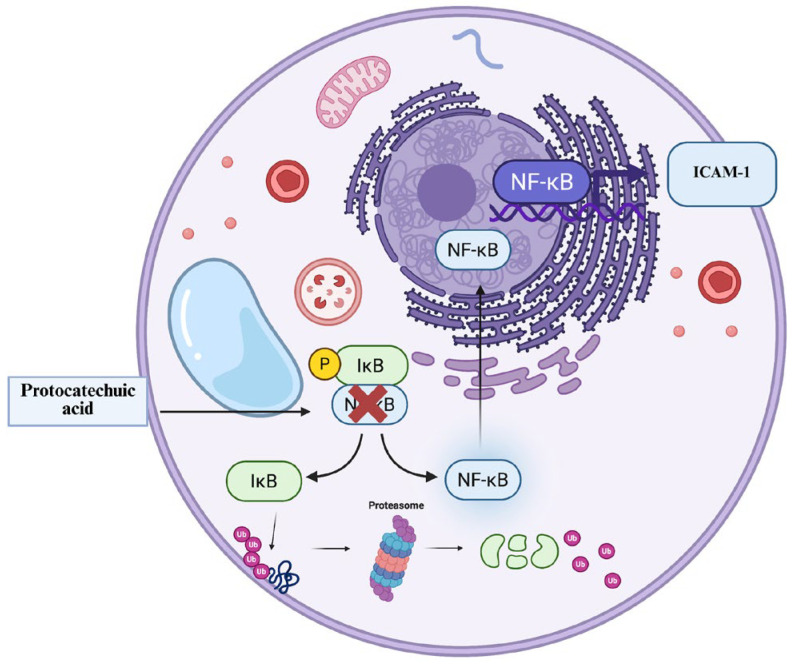
Protocatechuic acid-mediated NF-κB inhibition mechanism. High levels of NF-κB (nuclear factor kappa B) cause the inhibitor IKB (inhibitor of nuclear factor kappa B) to be degraded by the proteasome, releasing NF-κB so that it can translocate to the nucleus and carry out the transcription of proteins such as ICAM-1 (Intercellular Adhesion Molecule 1). In the presence of protocatechuic acid, NF-κB levels decrease, preventing the transcription of ICAM-1.

EET decreases MCF-7 invasion as a whole at 750 µg/mL and 96% at 500 µg/mL in MDA-MB-231. An investigation showed that protocatechuic acid decreases the levels of MMP-2 (matrix metalloproteinase-2) and MMP-9 (matrix metalloproteinase-9), which are zinc-dependent metalloproteinases responsible for degrading the extracellular matrix and allowing invasion; it also decreases the levels of the transcription factor NF-κB (Nucellular factor kappa B) [82]. By lowering NF-κB levels no stimulus allows IκB (inhibitor of nuclear factor kappa B) to phosphorylate, ubiquitinate, and be degraded by the proteasome, thus releasing NF-κB so that it can translocate to the nucleus [83] and allow the transcription of adhesion molecules such as ICAM-1 (Figure 10), which plays a role in adhesion and metastasis of cancer cells [84].

Nowadays, natural resources are used more frequently because compounds are obtained from them that can be used for the treatment of diseases and as therapeutic targets [85]. Chemotherapy has many adverse effects, including drug resistance. Cancer cells can develop resistance to cisplatin by decreasing the concentration by reducing its inflow through CRT1 (human creatine transporter 1) copper transporters and increasing its outflow through ATP7A (copper transporter P-type ATPase) transporters [86]. On the other hand, the main cause of paclitaxel resistance is cytoprotective autophagy [87] and is related to the cells’ attempt to counteract the impact of the cytotoxic compound, which is often necessary right at the start of treatment, for example, in triple-negative breast cancer (HCC38, HCC1143, Hs578T and MDA-MB-231). Guanylate-binding protein 5 (GBP5) increases sensitivity to this drug, and its removal consequently leads to increasing the required concentration of paclitaxel to kill cancer cells [88]. However, they take advantage of its ability to degrade specific proteins that cross the lysosomal membrane through lysosome-associated membrane protein type 2A (LAMP-2A) [89].

Plants are the major source of phytochemicals with anti-inflammatory, antioxidant and anticancer activity. The use of these for cancer treatment can improve the efficacy of chemotherapy by reducing toxicity in normal cells [90]. Therefore, the combined use of natural products with conventional drugs is common worldwide [91]. As can be seen in this study, the combination of the extract potentiated the cytotoxic effect of antineoplastic drugs cisplatin and paclitaxel. A study by Koraneekit et al. [90] showed that caffeic acid in combination with cisplatin can induce apoptosis by activating DNA damage. Sirota et al. [86] showed that caffeic acid can penetrate cells and inhibit GST (Glutathione S-transferase) and GSR (Glutathione Reductase) present at basal levels. By inhibiting the activity of these proteins, it causes cisplatin to increase its binding to DNA, causing apoptosis. Lin, C.-L. et al. [92] demonstrated that caffeic acid in combination with paclitaxel has synergistic effects, causing apoptosis and anti-proliferation in lung cancer cells via the NF-κB pathway.

## 4. Materials and Methods

### 4.1. Cell Culture

MCF-7 (ATCC^®^ HTB-22™) and MDA-MB-231 (ATCC^®^ HTB-26™) cell lines were cultured in DMEM GIBCO (Thermo Fisher Scientific Inc., Waltham, MA, USA) and Leibovitz-L15 medium, respectively. Both were supplemented with 10% GIBCO™ fetal bovine serum (FBS) (Thermo Fisher Scientific) and 1% GIBCO™ antibiotic–antimycotic (Thermo Fisher Scientific). Cultures were maintained under aseptic conditions at 37 °C and 5% CO_2_.

### 4.2. Preparation of the Extracts

One gram of the pulverized dry extract was dissolved in 5 mL of 80% ethanol, shaken for 30 min, and then filtered with Whatman No. 1 paper, gauging the supernatant obtained to the initial volume; this was called *Tabernaemonatana catharinensis* ethanol extract (EET). For cell assays, the solvent was evaporated and resuspended in culture medium and DMSO below 0.1%.

### 4.3. Ultra-Performance Liquid Chromatography with Time-of-Flight Mass Spectrometry (UPLC-Q-TOF/MS^E^) and Quantification of Compounds in Crude Extract by HPLC

EET was injected into the high-performance liquid chromatograph with Time-of-Flight Mass Spectrometry (UPLC-Q-TOF/MS Xevo) (model G2-XS, Waters, Milford, MA USA). All separations were performed with a Waters ACQUITY UHPLC^®^ HSS T3 (2.1 mm × 100 mm, particle size of 1.8 μm) at a column temperature of 40 °C and an autosampler temperature of 7 °C. The sample volume was 5 μL at each injection, and the liquid flow rate was 0.45 mL/min. The ionization parameters were as follows: cone voltage of 15 V and capillary voltage of 2.5 kV in negative mode. The desolvation temperature was set at 550 °C, while the ion source temperature was maintained at 120 °C. The desolvation gas (N_2_) flowed at 1000 L/h, while the cone gas (N_2_) flowed at 50 L/h. Data acquisition was performed using MassLynx software (V4.1. Waters Corporation, Milford, MA, USA). The UNIFI 1.8.0 platform (Waters, Manchester, UK) was used to analyze metabolites in the samples. The unique fragment patterns observed by mass spectrometry were compared with fragment ions documented in the literature and in data libraries such as PubChem (https://pubchem.ncbi.nlm.nih.gov/, accessed on 12 April 2025), Massbank (https://massbank.eu/MassBank/, accessed on 12 April 2025) and ChemSpider (https://www.chemspider.com/, accessed on 12 April 2025) by final metabolite identification [93,94,95]. HPLC-UV analysis was performed for the determination of the different compounds present in the 80% ethanol extract of *Tabernaemontana catharinensis*. This determination was performed by comparison with a standard curve. The concentrations used were 200, 150, 100, 75, 50, and 25 µg/mL. The standards and the extract were identified in an Agilent 1200 equipment, and a 25 × 4.6 cm Zorbax C18 analytical column was used. The stationary phase consisted of acetonitrile and water as the mobile phase. Ten µL of the samples were injected at a flow rate of 1 mL/min. Analyte detection was performed at 250, 254, and 280 nm.

### 4.4. Culture

After 90% confluence, the cell monolayer was trypsinized with 0.025% EDTA for 2 min. The cell suspension was centrifuged at 300× *g* for 5 min, and an aliquot of the suspension was taken and stained with 4% trypan blue to be counted in a Neubauer chamber.

### 4.5. Assays in 2D Cell Model

#### 4.5.1. D Cell Viability by SRB (Sulforhodamine B)

To determine the cytotoxicity of the extract, 20,000 cells of MCF-7 and MDA-MB-231 cell lines were seeded in 96-well plates. After 24 h of incubation, the extract was added after removal of the solvent and resuspended in DMSO (0.1%) and culture medium at different concentrations (3, 6.6, 33.3, 70, 100, 120, 140, 160, 180 µg/mL) and 13 µg/mL cisplatin for positive control in MCF-7 cell line and 3.3, 5, 6.6, 10, 15, 20, 25, 30, 33.3, 35, 66.6, and 100 µg/mL for MDA-MB-231 cell line and 13.65 µg/mL cisplatin and incubated for 24 h. A total of 100 µL of cold 10% TCA (Trichloroacetic Acid) was added to each well without removing the previous medium and incubated for 1 h. The plate was washed 4 times with distilled water, and 100 µL of 0.057% SRB (Sulforhodamine B) solution was added to each well. The plate was kept at room temperature for 30 min, then washed with 1% acetic acid and dried with paper towels, and 200 µL of 10 mM Tris solution was added to each well. The plate was shaken for 5 min and read on a Biotek SYNERGY H1 SYNERGY plate reader at 510 nm [96].

#### 4.5.2. Cell Cycle Assay in 2D Model in MCF-7 and MDA-MB-231 Cells, by Flow Cytometry

200,000 MCF-7 and MDA-MB-231 cells were seeded in a 3.5 cm 6-well plate (Nest^®^) (200,000/mL), subsequently synchronized in G0 phase with serum free culture medium for 24 h. To bring the cells out of G0 phase, culture medium supplemented with 10% FBS was added to the cells in the presence of 500 and 1000 µg/mL EET on one side and 13 and 13.65 µg/mL cisplatin on the other. Cells were trypsinized at 24 h, washed, and centrifuged to be fixed in 4% paraformaldehyde for 60 min. RnaseA and propidium iodide were added without exposure to light. DNA content was measured on a CytoFLEX S flow cytometer (Beckman Coulter), and a total of 20,000 events were acquired. Data analysis was performed in FlowJo^TM^Analysis Software version 10.10. The data are the result of three independent experiments performed in triplicate [97].

#### 4.5.3. Human Aldehyde Dehydrogenase 3A1 (ALDH3A1) Activity Assay

Human ALDH3A1 activity was determined according to the specifications of the Aldehyde Dehydrogenase Activity Assay Kit (Cayman Chemical, Ann Arbor, MI, USA). The concentration of 83.06 µg/mL of EET was used, and the absorbance reading was performed at 340 nm.

#### 4.5.4. Cyclin-Dependent Kinase 4 (CDK4) Activity Inhibition Assay

Inhibition of CDK4 activity was determined with the CDK4^®^ kit (BPS Bioscience, San Diego, CA, USA), following the manufacturer’s specifications. For the inhibition assay, concentrations of 8.3, 83.06, and 100 µg/mL of EET were used and incubated for 60 min. After that time, 50 μL of Kinase-Glo^®^ was added to each well and incubated for 15 min more, and then the luminescence was quantified with a microplate reader (SYNERGY H1, BioTek, Winooski, VT, USA).

#### 4.5.5. p53 Protein Transcription Assay

The p53 protein transcription assay was determined with p53 Transcription Factor Assay Kit (Cayman Chemical), following the manufacturer’s specifications. The concentrations of 83.06 µg/mL for MCF-7 and 8.3 µg/mL EET for MDA-MB-231 were used for the assay. Absorbance was measured in a microplate reader (SYNERGY H1, BioTek, Winooski, VT, USA) at 450 nm.

### 4.6. D Cell Model Assays

#### 4.6.1. Cell Viability by Plasma Membrane Integrity in 3D Culture of MCF-7 and MDA-MB-231 Cells

To determine the cytotoxicity of the extract on the cell lines in the 3D model, Sytox Green^®^ dye was used. A total of 1200 MCF-7 and MDA-MB-231 cells were seeded in 96-well ultra-low-adherent plates (Corning^®^). After spheroid formation, the cells were treated at 500, 1000, 1500 µg/mL of EET and 18.9 µg/mL of cisplatin for MCF-7 cell line; for MDA-MB-231 cell line the concentrations 74,140,280,420,560 µg/mL of EET and 20 µg/mL of cisplatin were used, incubated for 24 h. The dye was added, and fluorescence was measured in SYNERGY H1 microplate reader, Biotek.

#### 4.6.2. Quantification of ATP Levels in the 3D Model

CellTiter-Glo^®^3D (PROMEGA) was used to measure cell viability by ATP according to the manufacturer’s specifications. A total of 300 MCF-7 and MDA-MB-231 cells were seeded and incubated 24 h. The spheroids were treated with EET at concentrations 500, 600, 750 µg/mL and 18.9 µg/mL of cisplatin for MCF-7 and 280, 350, 500 µg/mL of EET and 20 µg/mL of cisplatin for MDA-MB-231, incubated for 24 h. CellTiter-Glo^®^ was added, and after addition of the reagent, the luminescence was quantified in a microplate reader (SYNERGY H1, BioTek, Winooski, VT, USA) for 30 min.

#### 4.6.3. Caspases -3/7, -8, and -9 Activity of MCF-7 and MDA-MB-231 Cells in 3D Model

To determine the effect of EET on caspase activity, the Caspase-Glo^®^, -3/7, -8, -9 kit (Promega Corp., Madison, WI, USA) was used, following the manufacturer’s specifications. A total of 300 MCF-7 and MDA-MB-231 cells were seeded per well, and after spheroid formation these were treated with EET at 500, 600, 750 µg/mL and 18.9 µg/mL of cisplatin and 280, 350, 500 µg/mL and 20 µg/mL of cisplatin, respectively. After the 24 h incubation period, Caspase-Glo^®^ reagent was added, and luminescence was quantified on a SYNERGY H1 microplate reader, Biotek.

#### 4.6.4. Tumor Invasion Assay in a 3D Model of MCF-7 and MDA-MB-231 Cells

For the invasion assay 1200 MCF-7 and MDA-MB-231 cells were seeded after spheroid formation (24 h). These were treated with EET at different concentrations (500, 600, 750 for MCF-7 and 280, 350, 500 µg/mL for MDA-MB-231) and 18.9 and 20 µg/mL of cisplatin, respectively, were incubated for 24 h. After that, 100 μL of Matrigel^®^ Matrix (Corning^®^) was added per well and then culture medium, DMEM for MCF-7 and Leibovitz-L15 for MDA-MB-231. Zeiss Axiovert 25 inverted microscope (Carl Zeiss, Oberkochen, Germany) was used to measure the area of invasion and processed with ImageJ software version 1.54g [98].

### 4.7. Statistical Analysis

The data of the experiments are expressed as mean, and the data were analyzed by one-way ANOVA with Graph Prism software version 10.4.1. Mean values and standard deviation of relative units of luminescence and absorbance by post hoc Turkey test (*p <* 0.0001). FlowJo^TM^version 10.10 software was used for cytometry analysis; the data are representative of three independent experiments, which were performed in triplicate.

## 5. Conclusions

The ethanolic extract of *Tabernaemontana catherinensis* (EET) shows promising therapeutic potential as an adjuvant in the treatment of breast cancer, exerting multiple antitumor effects on MCF-7 and MDA-MB-231 cell lines in both 2D and 3D models. Its ability to arrest the cell cycle in the G1 phase by inhibiting CDK4 and increasing p53, together with the induction of apoptosis through extrinsic and intrinsic pathways (activation of caspases -8, -9, and -3/7) and the decrease in ATP levels, suggests an efficient cytotoxic action. In addition, the inhibition of the invasive capacity of cells reinforces its role as an antimetastatic agent. Notably, EET potentiates the cytotoxic effects of conventional chemotherapeutic drugs such as cisplatin and paclitaxel, supporting its potential use as an adjuvant to improve the efficacy of current therapies and reduce possible adverse effects by decreasing the required doses. Together, these findings position EET as a promising natural alternative for the development of combined therapeutic strategies against breast cancer.

## Figures and Tables

**Table 1:** Identified compounds in EET by UPLC-Q-TOF/ MS^E^.

Identification	Tr	Formula	Measured *m*/*z*	Mass Error (ppm)	Fragmentation
Caffeic acid 3-glucoside	4.23	C_15_H_18_O_9_	341.0884	1.8	135.0436, 179.0340
Protocatechiuc acid	3.94	C_7_H_6_O_4_	153.0183	6.8	109.0237, 108.0195
Protocatechiuc acid 4-glucoside	2.55	C_13_H_16_O_9_	315.0726	1.5	109.0275, 153.0181
Chlorogenic acid	4.02	C_16_H_18_O_9_	353.0895	4.9	191.0555
Caffeic acid	4.84	C_9_H_8_O_4_	179.0348	−0.9	135.0438
Coniferyl aldehyde	5.67	C_10_H_10_O_3_	177.0551	−3.5	133.0280, 135.0432

**Table 2:** Quantification of compounds in ethanolic extract by HPLC.

Compound	µg/g Dry Sample
Chlorogenic acid	1248
Caffeic acid	118
Protocatechiuc acid	105

**Table 3:** Cell cycle arrest in G1 phase with EET treatment in the MCF-7 and MDA-MB-231 cell lines.

Treatment/ Phases	MCF-7			MDA-MB-231		
	G1	S	G2	G1	S	G2
Control	51.7 ± 3.04	3.7 ± 0.21	44.6 ± 1.30	56.7 ± 0.28	1.51 ± 0.12	41.75 ± 0.07
Starvation	64 ± 4.38	0.15 ± 0.11	35.85 ± 3.17	58 ± 0.49	5.65 ± 0.15	36.35 ± 3.87
Cisplatin 13/13.65 μg/mL	19.85 ± 3.46	79.4 ± 3.53	0.75 ± 0.07	16.2 ± 0.14	82.8 ± 0.14	1 ± 0
EET 500 μg/mL	70.3 ± 0.70	0.77 ± 0.04	28.2 ± 0.28	97.7 ± 3.25	2.3 ± 0.20	0 ± 0
EET 1000 μg/mL	86.1 ± 4.94	13.24 ± 0.88	0.66 ± 0.01	100 ± 0	0 ± 0	0 ± 0

**Table 4:** Effect of EET in combination with cisplatin and paclitaxel on the viability of MCF-7 and MDA-MB-231 cell lines in a 2D model.

Extract or Cisplatin/ Combination	MCF-7	MDA-MB-231	Extract or Paclitaxel/ Combination	MCF-7	MDA-MB-231
Cell Viability (%)			Cell Viability (%)		
Control	100	100	Control	100	100
½ IC_50_ EET	72	72	½ IC_50_ EET	73	73
IC_50_ EET	50	50	IC_50_ EET	51	51
½ IC_50_ Cisplatin	72	73	½ IC_50_ Paclitaxel	73	73
IC_50_ Cisplatin	50	50	IC_50_ Paclitaxel	50	50
½ IC_50_ EET + ½ IC_50_ Cisplatin	62	55	½ IC_50_ EET + ½ IC_50_ Paclitaxel	52	44
½ IC_50_ EET + IC_50_ Cisplatin	39	30	½ IC_50_ EET + IC_50_ Paclitaxel	29	34
IC_50_ EET + ½ IC_50_ Cisplatin	45	42	IC_50_ EET + ½ IC_50_ Paclitaxel	45	37
IC_50_ EET + IC_50_ Cisplatin	30	28	IC_50_ EET + IC_50_ Paclitaxel	19	28

Cisplatin IC_50_ in MCF-7: 13 ± 0.5 µg/mL; cisplatin IC_50_ in MDA-MB-231: 13.65 ± 0.1 µg/mL; paclitaxel IC_50_ in MCF-7: 6.1 ± 0.04 µg/mL; paclitaxel IC_50_ in MDA-MB-231: 0.96 ± 0.05 µg/mL; EET IC_50_ in MCF-7: 83.06 ± 0.6 µg/mL; EET IC_50_ in MDA-MB-231: 8.3 ± 0.57 µg/mL.

**Table 5:** Effect of EET in combination with cisplatin and paclitaxel on the viability of MCF-7 and MDA-MB-231 cell lines in a 3D model.

Extract or Cisplatin/ Combination	MCF-7	MDA-MB-231	Extract or Paclitaxel/ Combination	MCF-7	MDA-MB-231
Cell Viability (%)			Cell Viability (%)		
Control	100	100	Control	100	100
½ IC_50_ EET	72	73	½ IC_50_ EET	73	73
IC_50_ EET	50	51	IC_50_ EET	50	51
½ IC_50_ Cisplatin	74	75	½ IC_50_ Paclitaxel	74	74
IC_50_ Cisplatin	50	50	IC_50_ Paclitaxel	50	50
½ IC_50_ EET + ½ IC_50_ Cisplatin	67	66	½ IC_50_ EET + ½ IC_50_ Paclitaxel	62	67
½ IC_50_ EET + IC_50_ Cisplatin	39	42	½ IC_50_ EET + IC_50_ Paclitaxel	37	40
IC_50_ EET + ½ IC_50_ Cisplatin	42	45	IC_50_ EET + ½ IC_50_ Paclitaxel	42	43
IC_50_ EET + IC_50_ Cisplatin	37	38	IC_50_ EET + IC_50_ Paclitaxel	32	39

Cisplatin IC_50_ in MCF-7:18.9 ± 0.02 µg/mL; cisplatin IC_50_ in MDA-MB-231: 20 ± 0.1 µg/mL; paclitaxel IC_50_ in MCF-7: 17.34 ± 0.2 µg/mL; paclitaxel IC_50_ in MDA-MB-231: 5.93 ± 0.05 µg/mL; EET IC_50_ in MCF-7: 499.3 ± 0.71 µg/mL; EET IC_50_ in MDA-MB-231: 280 ± 0.10 µg/mL.

## Data Availability

Data are available upon request to the corresponding author.

## Supplementary Materials

The following supporting information can be downloaded at https://www.mdpi.com/article/10.3390/ijms26168111/s1.

## References

[1] McGrowder D.A., Miller F.G., Nwokocha C.R., Anderson M.S., Wilson-Clarke C., Vaz K., Anderson-Jackson L., Brown J. (2020). Medicinal Herbs Used in Traditional Management of Breast Cancer: Mechanisms of Action. Medicines.

[2] Globocan (2022). New Global Cancer Data. https://gco.iarc.fr/today/en/dataviz/pie?mode=cancer&group_populations=1.

[3] Xiong X., Zheng L.-W., Ding Y., Chen Y.-F., Cai Y.-W., Wang L.-P., Huang L., Liu C.-C., Shao Z.-M., Yu K.-D. (2025). Breast cancer: Pathogenesis and treatments. Signal Transduct. Target. Ther..

[4] Mahmoud A., Casciati A., Bakar Z.A., Hamzah H., Ahmad T.A.T., Noor M.H.M. (2023). The Detection of DNA Damage Response in MCF7 and MDA-MB-231 Breast Cancer Cell Lines after X-ray Exposure. Genome Integr..

[5] Wang X., Zhang H., Chen X. (2019). Drug resistance and combating drug resistance in cancer. Cancer Drug Resist..

[6] Wang H., Guo S., Kim S.-J., Shao F., Ho J.W.K., Wong K.U., Miao Z., Hao D., Zhao M., Xu J. (2021). Cisplatin prevents breast cancer metastasis through blocking early EMT and retards cancer growth together with paclitaxel. Theranostics.

[7] Rusia K., Madke B., Kashikar Y., Meghe S. (2023). Paclitaxel-Induced Cutaneous Lupus Erythematosus and Raynaud’s Phenomenon. Cureus.

[8] Abdelmaksoud N.M., Abulsoud A.I., Doghish A.S., Abdelghany T.M. (2023). From resistance to resilience: Uncovering chemotherapeutic resistance mechanisms; insights from established models. Biochim. Biophys. Acta (BBA)-Rev. Cancer.

[9] Iweala E.E.J., Amuji D.N., Nnaji F.C. (2024). Protein biomarkers for diagnosis of breast cancer. Sci. Afr..

[10] Valachis A., Biganzoli L., Christopoulou A., Fjermeros K., Fountzila E., Geisler J., Gomez-Bravo R., Karihtala P., Kosmidis P., Koutras A. (2024). Implementing geriatric assessment for dose optimization of CDK4/6 inhibitors in older breast cancer patients. Future Oncol..

[11] Li M., Cescon D.W. (2024). From Intractable to Treatable: Milestones and Horizons in the Management of HER2+ Breast Cancer. Can. Oncol. Today.

[12] Shalata W., Zolnoorian J., Migliozzi G., Jama A.A., Dudnik Y., Cohen A.Y., Meirovitz A., Yakobson A. (2023). Long-Lasting Therapeutic Response following Treatment with Pembrolizumab in Patients with Non-Small Cell Lung Cancer: A Real-World Experience. Int. J. Mol. Sci..

[13] Hayatou M.-U., Tembe E.A., Herve B., Borgia N.N., Fokunang C.N. (2023). Qualitative and Quantitative Phytochemical Characterization of Leaf Extracts of Mimosa pudica (Mimosaceae). J. Complement. Altern. Med. Res..

[14] Higashi B., De Almeida R.T.R., Pilau E.J., Gonçalves J.E., Gonçalves R.A.C., de Oliveira A.J.B. (2021). Metabolic profiling of monoterpenoid indole alkaloids from *Tabernaemontana catharinensis* (A. DC) latex by GC-MS. Phytochem. Lett..

[15] Rizo W.F., Ferreira L.E., Colnaghi V., Martins J.S., Franchi L.P., Takahashi C.S., Beleboni R.O., Marins M., Pereira P.S., Fachin A.L. (2013). Cytotoxicity and genotoxicity of coronaridine from *Tabernaemontana catharinensis* A.DC in a human laryngeal epithelial carcinoma cell line (Hep-2). Genet. Mol. Biol..

[16] Boligon A.A., Piana M., Schawnz T.G., Pereira R.P., Rocha J.B.T., Athayde M.L. (2014). Chromatographic Analysis and Antioxidant Capacity of *Tabernaemontana catharinensis*. Nat. Prod. Commun..

[17] Sari R., Conterno P., da Silva L.D., de Lima V.A., Oldoni T.L.C., Thomé G.R., Carpes S.T. (2020). Extraction of Phenolic Compounds from *Tabernaemontana catharinensis* Leaves and Their Effect on Oxidative Stress Markers in Diabetic Rats. Molecules.

[18] Mocanu M.-M., Nagy P., Szöllősi J. (2015). Chemoprevention of Breast Cancer by Dietary Polyphenols. Molecules.

[19] Rezaei-Seresht H., Cheshomi H., Falanji F., Movahedi-Motlagh F., Hashemian M., Mireskandari E. (2019). Cytotoxic activity of caffeic acid and gallic acid against MCF-7 human breast cancer cells: An *in silico* and *in vitro* study. Avicenna J. Phytomedicine.

[20] Guneidy R., Shokeer A., Abdel karim G.S.A., Saleh N., Zaki E. (2024). Effect of Protocatechuic Acid on Tamoxifen Efficacy and Oxidative Stress in Breast Cancer Cells: Implications for Combination Therapy. Egypt. J. Chem..

[21] Trujillo L., Bedoya J., Cortés N., Osorio E.H., Gallego J.-C., Leiva H., Castro D., Osorio E. (2023). Cytotoxic Activity of Amaryllidaceae Plants against Cancer Cells: Biotechnological, In Vitro, and In Silico Approaches. Molecules.

[22] Alam M., Ahmed S., Elasbali A.M., Adnan M., Alam S., Hassan M.I., Pasupuleti V.R. (2022). Therapeutic Implications of Caffeic Acid in Cancer and Neurological Diseases. Front. Oncol..

[23] Huang J., Xie M., He L., Song X., Cao T. (2023). Chlorogenic acid: A review on its mechanisms of anti-inflammation, disease treatment, and related delivery systems. Front. Pharmacol..

[24] Chiang H.-M., Chen C.-W., Chen C.-C., Wang H.-W., Jhang J.-H., Huang Y.-H., Wen K.-C. (2015). Role of Coffea arabica Extract and Related Compounds in Preventing Photoaging and Photodamage of the Skin. Coffee in Health and Disease Prevention.

[25] Song J., He Y., Luo C., Feng B., Ran F., Xu H., Ci Z., Xu R., Han L., Zhang D. (2020). New progress in the pharmacology of protocatechuic acid: A compound ingested in daily foods and herbs frequently and heavily. Pharmacol. Res..

[26] Khan H., Alam W., Alsharif K.F., Aschner M., Pervez S., Saso L. (2022). Alkaloids and Colon Cancer: Molecular Mechanisms and Therapeutic Implications for Cell Cycle Arrest. Molecules.

[27] Kabała-Dzik A., Rzepecka-Stojko A., Kubina R., Jastrzębska-Stojko Ż., Stojko R., Wojtyczka R., Stojko J. (2017). Comparison of Two Components of Propolis: Caffeic Acid (CA) and Caffeic Acid Phenethyl Ester (CAPE) Induce Apoptosis and Cell Cycle Arrest of Breast Cancer Cells MDA-MB-231. Molecules.

[28] Almalki S.G. (2023). The pathophysiology of the cell cycle in cancer and treatment strategies using various cell cycle checkpoint inhibitors. Pathol.-Res. Pract..

[29] Missihoun T.D., Kotchoni S.O., Bartels D. (2018). Aldehyde Dehydrogenases Function in the Homeostasis of Pyridine Nucleotides in *Arabidopsis thaliana*. Sci. Rep..

[30] Gelardi E.L.M., Colombo G., Picarazzi F., Ferraris D.M., Mangione A., Petrarolo G., Aronica E., Rizzi M., Mori M., La Motta C. (2021). A Selective Competitive Inhibitor of Aldehyde Dehydrogenase 1A3 Hinders Cancer Cell Growth, Invasiveness and Stemness In Vitro. Cancers.

[31] Duan J.-J., Cai J., Gao L., Yu S.-C. (2023). ALDEFLUOR activity, ALDH isoforms, and their clinical significance in cancers. J. Enzym. Inhib. Med. Chem..

[32] Qu Y., He Y., Yang Y., Li S., An W., Li Z., Wang X., Han Z., Qin L. (2020). ALDH3A1 acts as a prognostic biomarker and inhibits the epithelial mesenchymal transition of oral squamous cell carcinoma through IL-6/STAT3 signaling pathway. J. Cancer.

[33] Baker S.J., Poulikakos P.I., Irie H.Y., Parekh S., Reddy E.P. (2022). CDK4: A master regulator of the cell cycle and its role in cancer. Genes Cancer.

[34] Zhang W., Liu Y., Jang H., Nussinov R. (2024). CDK2 and CDK4: Cell Cycle Functions Evolve Distinct, Catalysis-Competent Conformations, Offering Drug Targets. JACS Au.

[35] Hernández Borrero L.J., El-Deiry W.S. (2021). Tumor suppressor p53: Biology, signaling pathways, and therapeutic targeting. Biochim. Biophys. Acta (BBA)-Rev. Cancer.

[36] Capuozzo M., Santorsola M., Bocchetti M., Perri F., Cascella M., Granata V., Celotto V., Gualillo O., Cossu A.M., Nasti G. (2022). p53: From Fundamental Biology to Clinical Applications in Cancer. Biology.

[37] Kepp O., Bezu L., Yamazaki T., Di Virgilio F., Smyth M.J., Kroemer G., Galluzzi L. (2021). ATP and cancer immunosurveillance. EMBO J..

[38] Mustafa M., Ahmad R., Tantry I.Q., Ahmad W., Siddiqui S., Alam M., Abbas K., Moinuddin, Hassan M.I., Habib S. (2024). Apoptosis: A Comprehensive Overview of Signaling Pathways, Morphological Changes, and Physiological Significance and Therapeutic Implications. Cells.

[39] Gupta G.P., Massagué J. (2006). Cancer Metastasis: Building a Framework. Cell.

[40] Gerstberger S., Jiang Q., Ganesh K. (2023). Metastasis. Cell.

[41] Torborg S.R., Li Z., Chan J.E., Tammela T. (2022). Cellular and molecular mechanisms of plasticity in cancer. Trends Cancer.

[42] Chou T.-C., Talalay P. (1984). Quantitative analysis of dose-effect relationships: The combined effects of multiple drugs or enzyme inhibitors. Adv. Enzym. Regul..

[43] Camponogara C., Casoti R., Brusco I., Piana M., Boligon A.A., Cabrini D.A., Trevisan G., Ferreira J., Silva C.R., Oliveira S.M. (2019). *Tabernaemontana catharinensis* leaves effectively reduce the irritant contact dermatitis by glucocorticoid receptor-dependent pathway in mice. Biomed. Pharmacother..

[44] Rosales P.F., Marinho F.F., Gower A., Chiarello M., Canci B., Roesch-Ely M., Paula F.R., Moura S. (2019). Bio-guided search of active indole alkaloids from *Tabernaemontana catharinensis*: Antitumour activity, toxicity in silico and molecular modelling studies. Bioorganic Chem..

[45] Boligon A.A., Schwanz T.G., Piana M., Bandeira R.V., Frohlich J.K., de Brum T.F., Zadra M., Athayde M.L. (2012). Chemical composition and antioxidant activity of the essential oil of *Tabernaemontana catharinensis* A. DC. leaves. Nat. Prod. Res..

[46] Da Silva Menecucci C., Mucellini K.L., de Oliveira M.M., Higashi B., de Almeida R.T.R., Porto C., Pilau E.J., Gonçalves J.E., Correia Gonçalves R.A., de Oliveira A.J.B. (2019). Latex from *Tabernaemontana catharinensis* (A. DC)—Apocynaceae: An alternative for the sustainable production of biologically active compounds. Ind. Crops Prod..

[47] Piana M., Boligon A.A., Brum T.F.D., Zadra M., Belke B.V., Froeder A.L.F., Frohlich J.K., Nunes L.T., Pappis L., Boligon A.A. (2014). Phytochemical analysis and antioxidant capacity of *Tabernaemontana catharinensis* A. DC. Fruits and branches. An. Acad. Bras. Ciências.

[48] Yin M.-C., Lin C.-C., Wu H.-C., Tsao S.-M., Hsu C.-K. (2009). Apoptotic Effects of Protocatechuic Acid in Human Breast, Lung, Liver, Cervix, and Prostate Cancer Cells: Potential Mechanisms of Action. J. Agric. Food Chem..

[49] Santos J.H., Hunakova L., Chen Y., Bortner C., Van Houten B. (2002). Cell Sorting Experiments Link Persistent Mitochondrial DNA Damage with Loss of Mitochondrial Membrane Potential and Apoptotic Cell Death. J. Biol. Chem..

[50] Hsu P.-H., Chen W.-H., Juan-Lu C., Hsieh S.-C., Lin S.-C., Mai R.-T., Chen S.-Y. (2021). Hesperidin and Chlorogenic Acid Synergistically Inhibit the Growth of Breast Cancer Cells via Estrogen Receptor/Mitochondrial Pathway. Life.

[51] Noratto G., Porter W., Byrne D., Cisneros-Zevallos L. (2009). Identifying Peach and Plum Polyphenols with Chemopreventive Potential against Estrogen-Independent Breast Cancer Cells. J. Agric. Food Chem..

[52] Xie C., Chan L., Pang Y., Shang Y., Cao W., Tuohan M., Deng Q., Wang Y., Zhao L., Wang W. (2024). Caffeic acid inhibits the tumorigenicity of triple-negative breast cancer cells through the FOXO1/FIS pathway. Biomed. Pharmacother..

[53] Yuan D., Wang J., Yan M., Xu Y. (2021). SOX2 as a prognostic marker and a potential molecular target in cervical cancer: A meta-analysis. Int. J. Biol. Markers.

[54] Tang Y., Tian W., Zheng S., Zou Y., Xie J., Zhang J., Li X., Sun Y., Lan J., Li N. (2023). Dissection of FOXO1-induced LYPLAL1-DT Impeding Triple Negative Breast Cancer Progression via Mediating hnRNPK/β-catenin Complex. Research.

[55] Agunloye O.M., Oboh G. (2018). Hypercholesterolemia, angiotensin converting enzyme and ecto-enzymes of purinergic system: Ameliorative properties of caffeic and chlorogenic acid in hypercholesterolemic rats. J. Food Biochem..

[56] Yegutkin G.G., Boison D. (2022). ATP and Adenosine Metabolism in Cancer: Exploitation for Therapeutic Gain. Pharmacol. Rev..

[57] Rosendahl A.H., Perks C.M., Zeng L., Markkula A., Simonsson M., Rose C., Ingvar C., Holly J.M.P., Jernström H. (2015). Caffeine and Caffeic Acid Inhibit Growth and Modify Estrogen Receptor and Insulin-like Growth Factor I Receptor Levels in Human Breast Cancer. Clin. Cancer Res..

[58] Jaganathan S.K. (2012). Growth Inhibition by Caffeic Acid, One of the Phenolic Constituents of Honey, in HCT 15 Colon Cancer Cells. Sci. World J..

[59] Ranjbary A.G., Bagherzadeh A., Sabbaghi S.S., Faghihi A., Karimi D.N., Naji S., Kardani M. (2023). Chlorogenic acid induces apoptosis and cell-cycle arrest in colorectal cancer cells. Mol. Biol. Rep..

[60] Pumiputavon K., Chaowasku T., Saenjum C., Osathanunkul M., Wungsintaweekul B., Chawansuntati K., Wipasa J., Lithanatudom P. (2017). Cell cycle arrest and apoptosis induction by methanolic leaves extracts of four Annonaceae plants. BMC Complement. Altern. Med..

[61] Chinnasamy A., Jayaprakash V., Padmanaban D., Sekar N., Valayapathi R., Azhagudurai A., Ethiraj S. (2024). Effect of crude ethanolic seed extract from Mucuna pruriens on proliferation, apoptosis, and cell cycle arrest in gastric adenocarcinoma (AGS) cells. Future J. Pharm. Sci..

[62] Chen Y., Yan H., Yan L., Wang X., Che X., Hou K., Yang Y., Li X., Li Y., Zhang Y. (2023). Hypoxia-induced ALDH3A1 promotes the proliferation of non-small-cell lung cancer by regulating energy metabolism reprogramming. Cell Death Dis..

[63] Sládek N., Kollander R., Sreerama L., Kiang D. (2002). Cellular levels of aldehyde dehydrogenases (ALDH1A1 and ALDH3A1) as predictors of therapeutic responses to cyclophosphamide-based chemotherapy of breast cancer: A retrospective study. Cancer Chemother. Pharmacol..

[64] Gupta N., Dogra S., Dimri K., Pandey A.K., Jose J.S., Punia R. (2024). Metaplastic breast cancer: Experience with ifosfamide based chemotherapy. Curr. Probl. Cancer.

[65] Voulgaridou G.-P., Kiziridou M., Mantso T., Chlichlia K., Galanis A., Koukourakis M.I., Franco R., Panayiotidis M.I., Pappa A. (2016). Aldehyde dehydrogenase 3A1 promotes multi-modality resistance and alters gene expression profile in human breast adenocarcinoma MCF-7 cells. Int. J. Biochem. Cell Biol..

[66] Ye F., Qiu Y., Li L., Yang L., Cheng F., Zhang H., Wei B., Zhang Z., Sun L., Bu H. (2015). The Presence of EpCAM-/CD49f+Cells in Breast Cancer Is Associated with a Poor Clinical Outcome. J. Breast Cancer.

[67] Stengel K.R., Thangavel C., Solomon D.A., Angus S.P., Zheng Y., Knudsen E.S. (2009). Retinoblastoma/p107/p130 Pocket Proteins. J. Biol. Chem..

[68] Huang J., Zheng L., Sun Z., Li J. (2022). CDK4/6 inhibitor resistance mechanisms and treatment strategies (Review). Int. J. Mol. Med..

[69] Liang Y., Feng G., Wu L., Zhong S., Gao X., Tong Y., Cui W., Qin Y., Xu W., Xiao X. (2019). Caffeic acid phenethyl ester suppressed growth and metastasis of nasopharyngeal carcinoma cells by inactivating the NF-κB pathway. Drug Des. Dev. Ther..

[70] Wadhwa R., Nigam N., Bhargava P., Dhanjal J.K., Goyal S., Grover A., Sundar D., Ishida Y., Terao K., Kaul S.C. (2016). Molecular Characterization and Enhancement of Anticancer Activity of Caffeic Acid Phenethyl Ester by γ Cyclodextrin. J. Cancer.

[71] Razak N.A., Abu N., Ho W.Y., Zamberi N.R., Tan S.W., Alitheen N.B., Long K., Yeap S.K. (2019). Cytotoxicity of eupatorin in MCF-7 and MDA-MB-231 human breast cancer cells via cell cycle arrest, anti-angiogenesis and induction of apoptosis. Sci. Rep..

[72] Lin Y., Wu Y.S., Chao M., Yang D., Liu C., Tseng J., Chen Y. (2024). An alleviative effect of *Lonicerae japonicae* flos water extract against liver fibrogenesis in vitro and in vivo. Environ. Toxicol..

[73] Abdel-Razek M.A.M., Abdelwahab M.F., Mohamad S.A., Abou-Zied H.A., Abdelmohsen U.R., Hamed A.N.E. (2025). Cytotoxic Potential and Metabolomic Profiling of *Solanum lycopersicum* Roots Extract and Their Nanocrystals: An *In Silico* Approach. Integr. Cancer Ther..

[74] Kabalan Y., Matulewicz K., Tylkowski B., Woźniak-Budych M., Staszak K., Montané X., Bajek A. (2025). Investigation of Anti-Cancer Properties of Nano-Encapsulated Ciprofloxacin Using 3D Cancer Cell Spheroids as Tumour Models. Int. J. Mol. Sci..

[75] Cordeiro S., Oliveira B.B., Valente R., Ferreira D., Luz A., Baptista P.V., Fernandes A.R. (2024). Breaking the mold: 3D cell cultures reshaping the future of cancer research. Front. Cell Dev. Biol..

[76] Ingber D.E. (2022). Human organs-on-chips for disease modelling, drug development and personalized medicine. Nat. Rev. Genet..

[77] Wagner M., Wiig H. (2015). Tumor Interstitial Fluid Formation, Characterization, and Clinical Implications. Front. Oncol..

[78] Winkler J., Abisoye-Ogunniyan A., Metcalf K.J., Werb Z. (2020). Concepts of extracellular matrix remodelling in tumour progression and metastasis. Nat. Commun..

[79] Sandra F., Putri J., Limen H., Sarizta B. (2021). Caffeic Acid Inhibits RANKL and TNFa-induced Osteoclastogenesis by Targeting TAK1-p44/42 MAPK. Indones. Biomed. J..

[80] Yue J., López J.M. (2020). Understanding MAPK Signaling Pathways in Apoptosis. Int. J. Mol. Sci..

[81] Orlowski R.Z., Small G.W., Shi Y.Y. (2002). Evidence That Inhibition of p44/42 Mitogen-activated Protein Kinase Signaling Is a Factor in Proteasome Inhibitor-mediated Apoptosis. J. Biol. Chem..

[82] Lin H.-H., Chen J.-H., Chou F.-P., Wang C.-J. (2010). Protocatechuic acid inhibits cancer cell metastasis involving the down-regulation of Ras/Akt/NF-κB pathway and MMP-2 production by targeting RhoB activation. Br. J. Pharmacol..

[83] López-Bojorquez L.N. (2004). La regulación del factor de transcripción NF-κB. Un mediador molecular en el proceso inflamatorio. Rev. Investig. Clínica.

[84] Qiu Z., Wang Y., Zhang Z., Qin R., Peng Y., Tang W., Xi Y., Tian G., Zhang Y. (2022). Roles of intercellular cell adhesion molecule-1 (ICAM-1) in colorectal cancer: Expression, functions, prognosis, tumorigenesis, polymorphisms and therapeutic implications. Front. Oncol..

[85] Wainwright C.L., Teixeira M.M., Adelson D.L., Buenz E.J., David B., Glaser K.B., Harata-Lee Y., Howes M.-J.R., Izzo A.A., Maffia P. (2022). Future directions for the discovery of natural product-derived immunomodulating drugs: An IUPHAR positional review. Pharmacol. Res..

[86] Sirota R., Gibson D., Kohen R. (2017). The timing of caffeic acid treatment with cisplatin determines sensitization or resistance of ovarian carcinoma cell lines. Redox Biol..

[87] LIU S., LI X. (2015). Autophagy inhibition enhances sensitivity of endometrial carcinoma cells to paclitaxel. Int. J. Oncol..

[88] Škubník J., Pavlíčková V.S., Ruml T., Rimpelová S. (2023). Autophagy in cancer resistance to paclitaxel: Development of combination strategies. Biomed. Pharmacother..

[89] Macho-González A., Sánchez-Muniz F.J. (2022). Autophagy, a key cellular cleansing system for health. A visit to the 2016 Nobel Prize in Physiology or Medicine. J. Negat. No Posit. Results.

[90] Koraneekit A., Limpaiboon T., Sangka A., Boonsiri P., Daduang S., Daduang J. (2018). Synergistic effects of cisplatin-caffeic acid induce apoptosis in human cervical cancer cells via the mitochondrial pathways. Oncol. Lett..

[91] Li C., Jia W.-W., Yang J.-L., Cheng C., Olaleye O.E. (2022). Multi-compound and drug-combination pharmacokinetic research on Chinese herbal medicines. Acta Pharmacol. Sin..

[92] Lin C.-L., Chen R.-F., Chen J.Y.-F., Chu Y.-C., Wang H.-M., Chou H.-L., Chang W.-C., Fong Y., Chang W.-T., Wu C.-Y. (2012). Protective Effect of Caffeic Acid on Paclitaxel Induced Anti-Proliferation and Apoptosis of Lung Cancer Cells Involves NF-κB Pathway. Int. J. Mol. Sci..

[93] Hou Y.-F., Bai L., Guo S., Hu J.-B., Zhang S.-S., Liu S.-J., Zhang Y., Li S., Ho C.-T., Bai N.-S. (2023). Nontargeted metabolomic analysis of four different parts of Actinidia arguta by UPLC-Q-TOF-MSE. Food Res. Int..

[94] LIU P.-P., SHAN G.-S., ZHANG F., CHEN J.-N., JIA T.-Z. (2018). Metabolomics analysis and rapid identification of changes in chemical ingredients in crude and processed Astragali Radix by UPLC-QTOF-MS combined with novel informatics UNIFI platform. Chin. J. Nat. Med..

[95] Vannabhum M., Ziangchin N., Thepnorarat P., Akarasereenont P. (2023). Metabolomic analysis of Thai Herbal Analgesic Formula based on ultra-high-performance liquid chromatography-quadrupole time-of-flight mass spectrometry. Heliyon.

[96] Vichai V., Kirtikara K. (2006). Sulforhodamine B colorimetric assay for cytotoxicity screening. Nat. Protoc..

[97] Gomes D., Telles C., Costa M., Almeida-Lima J., Costa L., Keesen T., Rocha H. (2015). Methanolic Extracts from Brown Seaweeds Dictyota cilliolata and Dictyota menstrualis Induce Apoptosis in Human Cervical Adenocarcinoma HeLa Cells. Molecules.

[98] Martínez-Rodríguez O.P., González-Torres A., Álvarez-Salas L.M., Hernández-Sánchez H., García-Pérez B.E., Thompson-Bonilla M.d.R., Jaramillo-Flores M.E. (2021). Effect of naringenin and its combination with cisplatin in cell death, proliferation and invasion of cervical cancer spheroids. RSC Adv..

