# Supplementary material for: Anticancer Activity of Ethanolic Extract of Tabernaemontana catharinensis in Breast Cancer Lines MCF-7 and MDA-MB-231
Source: Int J Mol Sci. 2025 Aug 21;26(16):8111. doi: 10.3390/ijms26168111 (PMC12386794; doi:10.3390/ijms26168111)
Supplement: Supplementary file 1 [file ijms-26-08111-s001.zip › ijms-3796939-supplementary.pdf]

Table S1. Combination indices (CI) calculated for all combinations using the Chou–Talalay method.

Model	Drug	Line	Fracciones	Viability %	Remaining fraction	CI	Interpretation
2D	Cisplatin	MCF-7	0.5+0.5	62.000	0.380	1.432	Antagonism
2D	Cisplatin	MCF-7	0.5+1.0	39.000	0.610	1.080	Antagonism
2D	Cisplatin	MCF-7	1.0+0.5	45.000	0.550	1.295	Antagonism
2D	Cisplatin	MCF-7	1.0+1.0	30.000	0.700	1.074	Antagonism
2D	Cisplatin	MDA-MB-231	0.5+0.5	55.000	0.450	1.154	Antagonism
2D	Cisplatin	MDA-MB-231	0.5+1.0	30.000	0.700	0.814	Synergy
2D	Cisplatin	MDA-MB-231	1.0+0.5	42.000	0.580	1.193	Antagonism
2D	Cisplatin	MDA-MB-231	1.0+1.0	28.000	0.720	1.018	Additive
2D	Paclitaxel	MCF-7	0.5+0.5	52.000	0.480	1.057	Antagonism
2D	Paclitaxel	MCF-7	0.5+1.0	29.000	0.710	0.804	Synergy
2D	Paclitaxel	MCF-7	1.0+0.5	45.000	0.550	1.304	Antagonism
2D	Paclitaxel	MCF-7	1.0+1.0	19.000	0.810	0.728	Synergy
2D	Paclitaxel	MDA-MB-231	0.5+0.5	44.000	0.560	0.845	Synergy
2D	Paclitaxel	MDA-MB-231	0.5+1.0	34.000	0.660	0.945	Synergy
2D	Paclitaxel	MDA-MB-231	1.0+0.5	37.000	0.630	1.035	Additive
2D	Paclitaxel	MDA-MB-231	1.0+1.0	28.000	0.720	1.036	Additive
3D	Cisplatin	MCF-7	0.5+0.5	67.000	0.330	1.618	Antagonism
3D	Cisplatin	MCF-7	0.5+1.0	39.000	0.610	1.110	Antagonism
3D	Cisplatin	MCF-7	1.0+0.5	42.000	0.580	1.202	Antagonism
3D	Cisplatin	MCF-7	1.0+1.0	37.000	0.630	1.393	Antagonism
3D	Cisplatin	MDA-MB-231	0.5+0.5	66.000	0.340	1.602	Antagonism
3D	Cisplatin	MDA-MB-231	0.5+1.0	42.000	0.580	1.227	Antagonism
3D	Cisplatin	MDA-MB-231	1.0+0.5	45.000	0.550	1.340	Antagonism
3D	Cisplatin	MDA-MB-231	1.0+1.0	38.000	0.620	1.464	Antagonism
3D	Paclitaxel	MCF-7	0.5+0.5	62.000	0.380	1.395	Antagonism
3D	Paclitaxel	MCF-7	0.5+1.0	37.000	0.630	1.048	Additive
3D	Paclitaxel	MCF-7	1.0+0.5	42.000	0.580	1.202	Antagonism
3D	Paclitaxel	MCF-7	1.0+1.0	32.000	0.680	1.198	Antagonism
3D	Paclitaxel	MDA-MB-231	0.5+0.5	67.000	0.330	1.670	Antagonism
3D	Paclitaxel	MDA-MB-231	0.5+1.0	40.000	0.600	1.152	Antagonism
3D	Paclitaxel	MDA-MB-231	1.0+0.5	43.000	0.570	1.263	Antagonism
3D	Paclitaxel	MDA-MB-231	1.0+1.0	39.000	0.610	1.496	Antagonism

CI < 1 indicates synergy, CI ≈ 1 indicates additive effect, and CI > 1 indicates antagonism.

Isobolograms of the 2D model

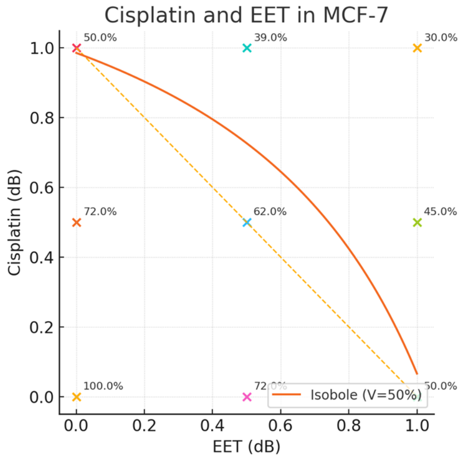

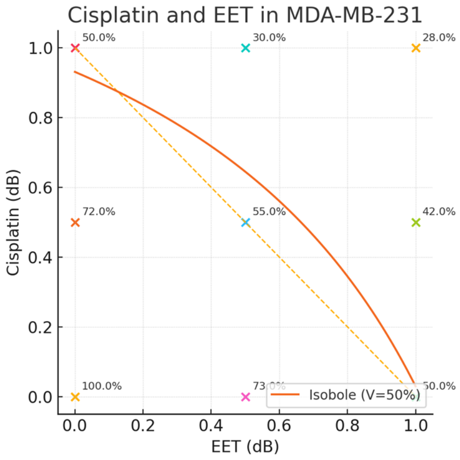

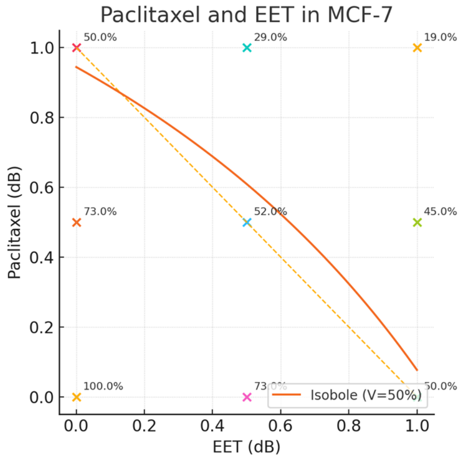

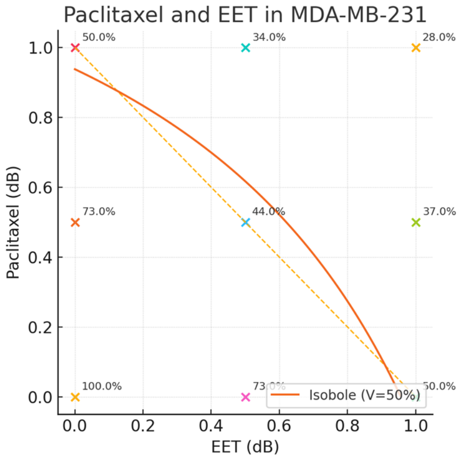

Isobolograms of the 3D model

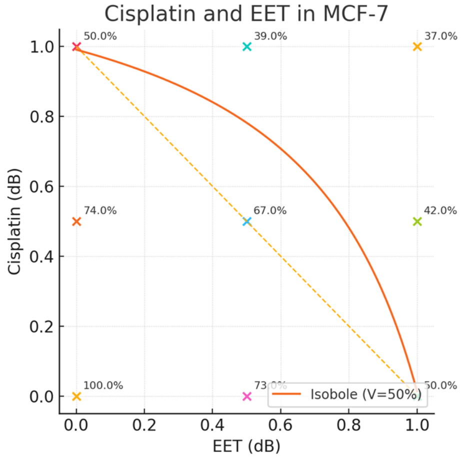

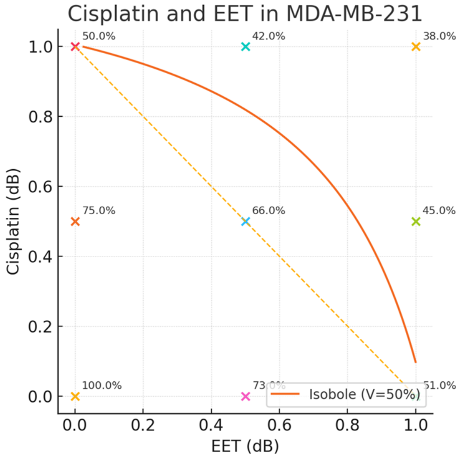

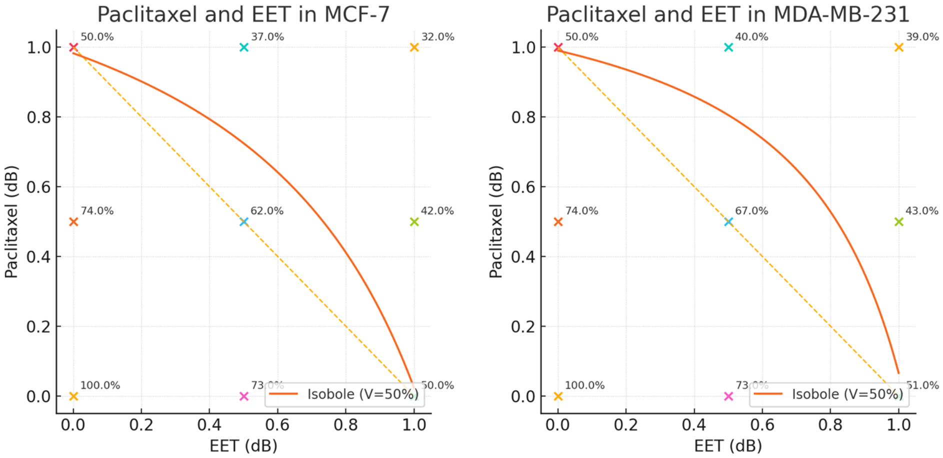

Paclitaxel and EET in MCF-7
Paclitaxel (dB)
EET (dB)
50.0%
37.0%
32.0%
74.0%
62.0%
42.0%
100.0%
50.0%
Isobole (V=50%)
Paclitaxel and EET in MDA-MB-231
Paclitaxel (dB)
EET (dB)
50.0%
40.0%
39.0%
74.0%
67.0%
43.0%
100.0%
51.0%
Isobole (V=50%)
